# Clinical approaches to treating papillary squamous cell carcinoma of the uterine cervix

**DOI:** 10.1186/1471-2407-14-784

**Published:** 2014-10-27

**Authors:** Michikazu Nagura, Masafumi Koshiyama, Noriomi Matsumura, Aki Kido, Tsukasa Baba, Kaoru Abiko, Junzo Hamanishi, Ken Yamaguchi, Yoshiki Mikami, Ikuo Konishi

**Affiliations:** Department of Gynecology and Obstetrics, Kyoto University, Graduate School of Medicine, 54 Shogoin Kawahara-cho, Sakyo-ku, Kyoto, 606-8507 Japan; Department of Diagnostic Imaging and Nuclear Medicine, Kyoto University, Graduate School of Medicine, Kyoto, Japan; Department of Diagnostic Pathology, Kyoto University, Graduate School of Medicine, Kyoto, Japan

**Keywords:** Papillary squamous cell carcinoma, PSCC, Uterine cervix, MRI

## Abstract

**Background:**

Papillary squamous cell carcinoma (PSCC) of the uterine cervix is difficult to diagnose due to its rarity and limited data regarding its clinical behavior. We attempted to assess the degree of stromal invasion using magnetic resonance imaging (MRI) and evaluate possible treatments for this lesion in view of its clinical behavior.

**Methods:**

We analyzed 28 cases of PSCC diagnosed on the colposcopic selective biopsies. We studied the rate of accuracy of diagnoses of the colposcopic selective biopsies compared with the final diagnoses, and compared the rate of stromal invasion between the MRI and pathological findings while focusing on surgical methods and the clinical prognosis.

**Results:**

Of the 28 patients, only 12 exhibited true PSCC. The other 16 patients were ultimately diagnosed with SCC or adenosquamous carcinoma based on the finding of the surgical specimens and exhibited relatively poor prognoses. Among the 12 true PSCC cases, the rate of diagnostic accuracy of stromal invasion (with or without) was only 58% (7/12) on the colposcopic selective biopsies. However, we were able to predict the presence of stromal invasion (microscopic borderline: approximately 3 mm) before surgery using MRI. None of the 10 patients treated with radical surgery displayed lymph node metastases. In addition, all 12 study patients exhibited no recurrence (mean: 49 months) and survived.

**Conclusions:**

MRI can be used to detect preinvasive and microinvasive disease before surgery. It is possible to select a less invasive surgical method than radical surgery in cases of preinvasive and microinvasive PSCC in view of the indolent clinical behavior of this disease.

**Electronic supplementary material:**

The online version of this article (doi:10.1186/1471-2407-14-784) contains supplementary material, which is available to authorized users.

## Background

Papillary squamous cell carcinoma (PSCC) of the uterine cervix is a very rare variant and poorly documented subtype of squamous cell carcinoma (SCC). Its incidence has been reported to be 1.6% of cervical carcinomas [1].

PSCC is not easily diagnosed based on the colposcopic selective biopsies and physicians are often unable to recognize the tumor depth [2]. The colposcopic selective biopsies may detect PSCC-like lesions; however, these masses may account for small portions of the main tumor whose major component is SCC. Therefore, in patients treated with radiation therapy, there is a risk that a diagnosis of PSCC obtained on a colposcopic selective biopsy will remain the final diagnosis after treatment. The use of radical hysterectomy allows physicians to evaluate the entire tumor and make an exact diagnosis.

PSCC grows superficially with wart-like or exophytic features. Lesions with a gross appearance including carcinomatous or papillary growth on the cervix are diagnosed as invasive carcinoma based on the International Federation of Gynecology and Obstetrics (FIGO) staging (NCCN Guidelines, version 2.2013). In such cases, the recommended treatment is radical hysterectomy with lymphadenectomy or radiation therapy (NCCN Guidelines, version 2.2013). However, these lesions may also contain elements of preinvasive and microinvasive disease. To our knowledge, there are no previous reports of lymph node metastases in patients with preinvasive and microinvasive disease. The clinical behavior of this entity is not well understood. In addition, there are no reports of the prognosis of minimally invasive surgery in these patients. Therefore, it is difficult to identify the best treatment strategy for PSCC.

In this study, we performed surgical resection in all cases of PSCC to make an exact diagnosis. We then considered possible improvements to management strategies. In this manner, the current manuscript provides the findings of our observational and diagnostic accuracy study.

## Methods

Patients with PSCC and SCC treated at Kyoto University Hospital within a 10-year period (2003 ~ 2012) were assessed in this study. The study protocol was approved by the ethics committee of Kyoto University Hospital (No. G288), and all patients provided their written informed consent prior to study entry. A total of 699 SCC patients were diagnosed on the colposcopic selective biopsies during the 10-year study period. Among them, we found 28 surgical patients who were initially diagnosed with PSCC of the uterine cervix on the colposcopic selective biopsy. Primary surgery was performed to obtain the final diagnosis after having obtained informed consent from each patient. We divided the 28 patients into true PSCC patients, who were diagnosed with PSCC on both the colposcopic selective biopsy and surgical specimens, and false PSCC patients, who were initially diagnosed with PSCC on the colposcopic selective biopsy, and whose diagnosis ultimately changed on the surgical specimens to a condition other than PSCC. In our hospital, the microscopic diagnosis was initially made in the outpatient clinic using a colposcopic selective biopsy. In some cases, we performed conization to obtain a more precise microscopic diagnosis. We used magnetic resonance imaging (MRI) in all patients with uterine cervical cancer to evaluate the tumor depth and degree of tumor spread preoperatively. We then compared the tumor depth (particularly the degree of stromal invasion) on MRI with the microscopic tumor depth observed in the surgical specimens. We subsequently examined the pathological findings on many glass slides and studied the depth of tumor invasion and metastasis. In addition, we used computed tomography (CT) scans to determine the presence of lymph node or distant metastasis. Clinical staging was performed according to the FIGO staging system. The rate of recurrence and length of survival were followed until 2013 to evaluate the prognosis. We also assessed treatment strategies for PSCC and evaluated the presence of lymph node metastases and patients’ prognoses. Finally, we considered possible improvements to management strategies, particularly with regard to selecting minimally invasive surgery for patients with preinvasive and microinvasive disease desiring fertility.

## Results

Over the 10-year study period, 28 cases of PSCC were diagnosed based on the colposcopic selective biopsies prior to treatment. Only 12 (43%) of the 28 patients were also diagnosed with PSCC after surgery. The diagnoses in the other 16 (57%) cases were ultimately changed to SCC or adenosquamous carcinoma based on the results of the surgical specimens.

**Figure 1:**
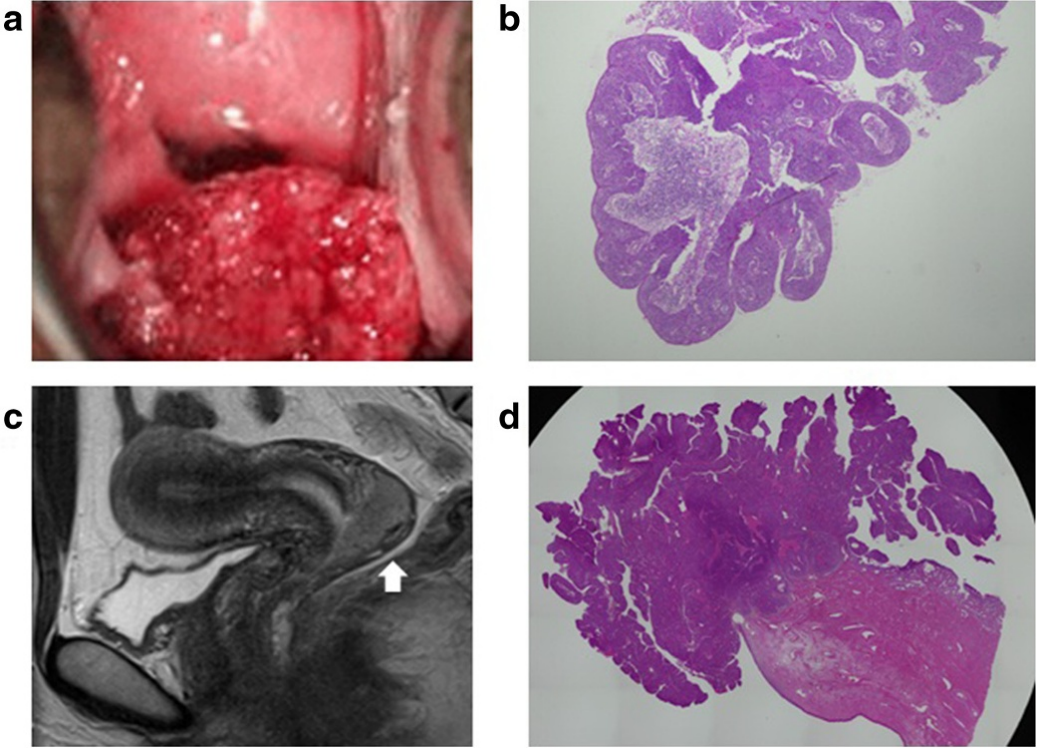
**A representative true PSCC case (Patient No. 10). a**. Patient No. 10 had a cauliflower-shaped lesion on the posterior lip of the cervix (gross observation). **b**. Papillary projections of atypical epithelia were found on the colposcopic selective biopsy in Patient No. 10. **c**. Patient No. 10 had a 41-mm-sized exophytic lesion on the surface of the posterior lip of the cervix (T2-weighted sagittal MRI). **d**. The surgical specimen of Patient No. 10 showed an exophytic papillary lesion, while the depth of stromal invasion was not great (H and E, low-power view).

The clinical features of the 12 true PSCC cases are summarized in Additional file 1: Table S1. The mean age of these 12 patients was 49.6 years, with a range of 30 to 69 years. There was one (8%) patient with carcinoma in situ (CIS), seven (58%) patients with stage IB1 disease, two (17%) patients with stage IB2 disease, one (8%) patient with stage IIA1 disease and one (8%) patient with stage IIB disease. All of the 12 PSCC patients were alive at the time of the last contact, with no episodes of recurrence over a mean follow-up of 49 months (range: 7 to 82 months). A representative true PSCC case (Patient No.10) is presented in Figure 1a-d.

**Figure 2:**
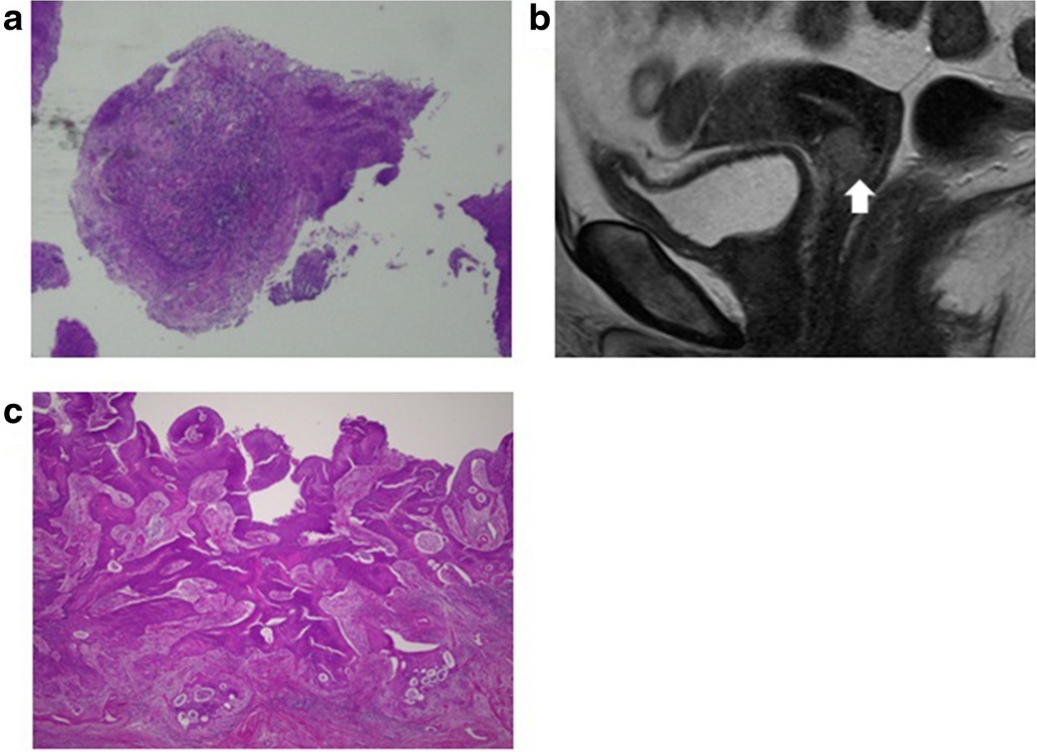
**A representative false PSCC case (Patient No. 19). a**. Several small crowds of malignant cells appeared PSCC-like on the colposcopic selective biopsy in Patient No. 19. **b**. Patient No.19 had a 13-mm-sized invasive lesion on the anterior lip of the cervix (T2-weighted sagittal MRI). **c**. The surgical specimen of Patient No. 19 showed destructive invasion extending to deep stroma with lymphovascular space invasion, which was predominant over the papilla-like exophytic growth (H and E, 20 X). Consequentially, we obtained a diagnosis of non-keratinizing SCC.

The clinical features of the 16 false PSCC patients are summarized in Additional file 2: Table S2. The mean age of these 16 patients was 51.8 years, with a range of 34 to 68 years. There was one (6%) patient with stage IA2 disease, six (38%) patients with stage IB1 disease, three (19%) patients with stage IB2 disease, one (6%) patient with stage IIA1 disease and five (31%) patients with stage IIB disease. A representative false PSCC case (Patient No.19) is presented in Figure 2a-c. Histologically, two cases were diagnosed as microinvasive SCC, 13 cases were diagnosed as non-keratinizing SCC, and one case was diagnosed as adenosquamous carcinoma based on the results of the surgical specimens. Twelve (75%) of the 16 false PSCC patients were alive at the time of the last contact, with no episodes of recurrence over a mean follow-up of 50 months (range: 17 to 120 months). However, four (25%) of the 16 false PSCC patients (at stage IB2, IB2, IIB and IIB) developed recurrence, and three (19%) patients (at stage IB2, IB2 and IIB) died.

**Figure 3:**
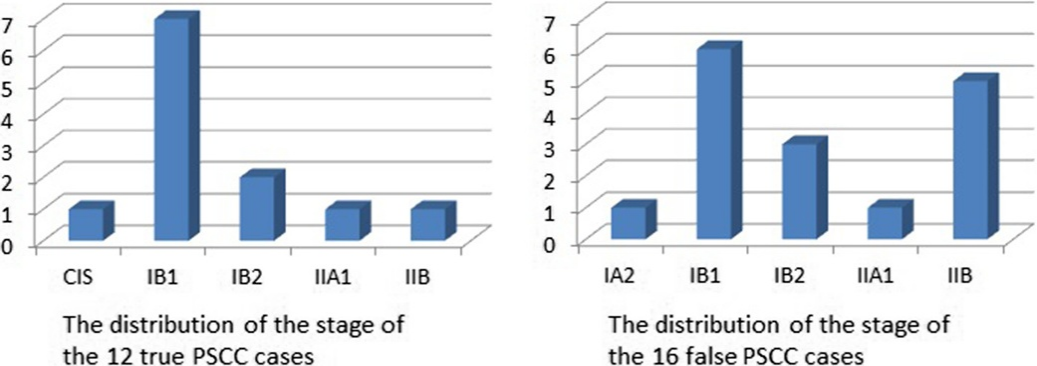
**The distribution of the stage of the 12 true PSCC cases and the 16 false PSCC cases.** The former was associated with an early stage (stageIB1), whereas the latter was associated with a more advanced stage (stage IB1 ~ IIB).

The distribution of stage in the 12 true PSCC cases and 16 false PSCC cases is presented in Figure 3. The former group exhibited a trend toward an early stage of disease (stage IB1), whereas the latter group was associated with more advanced stages (stage IB1 ~ IIB).

**Figure 4:**
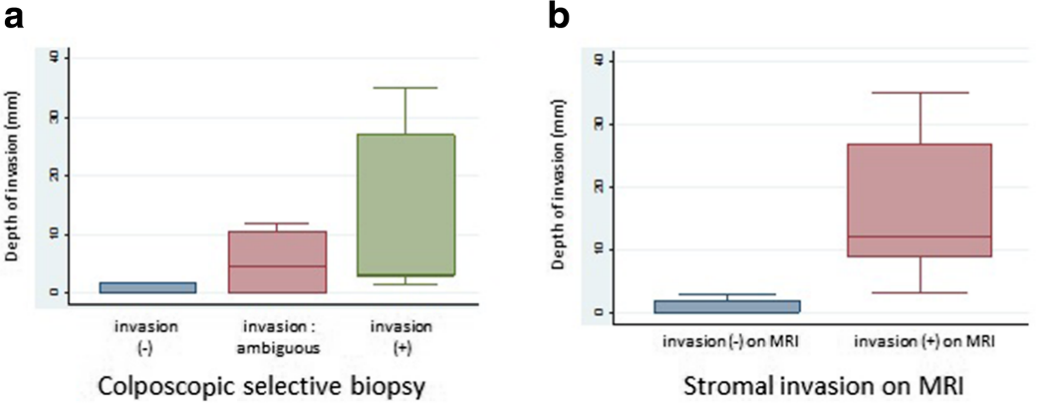
**The relationship between the predictive invasion and the final depth of the invasion. a**. The relationship between the stromal invasion (with, without and unclear) on the colposcopic selective biopsies and the final depth of the invasion on the surgical specimens. Four (33%) PSCC cases involving stromal invasion were ambiguous on the colposcopic selective biopsies. Ultimately, two (50%) of these patients exhibited no stromal invasion and two (50%) exhibited stromal invasion on the surgical specimens. **b**. The relationship between the stromal invasion (with or without) on MRI and the final depth of the invasion on the surgical specimens. The two groups in which invasion was or was not detected on MRI did not overlap.

Three (25%) of the 12 patients with PSCC did not exhibit stromal invasion on the colposcopic selective biopsies (Additional file 3: Table S3). However, one of these three patients displayed a depth of invasion of 1.8 mm on the surgical specimens. Five (42%) of the 12 patients with PSCC were found to have stromal invasion on the colposcopic selective biopsies; all of these five patients also demonstrated stromal invasion on the surgical specimens. However, four (33%) PSCC cases involving stromal invasion were ambiguous on the colposcopic selective biopsies. Among the 12 true PSCC cases, the rate of diagnostic accuracy of stromal invasion was only 58% (7/12) on the colposcopic selective biopsies (Additional file 3: Table S3). The range of final depth in this category was wide (Figure 4a). As a whole, it was difficult to determine whether PSCC was associated with stromal invasion using the colposcopic selective biopsies.

**Figure 5:**
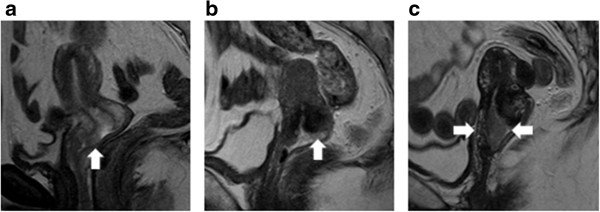
**The relationship between the MRI findings before surgery and the final depth of tumor invasion. a**. Patient No.6. On T2-weighted sagittal images, a small mass of high intensity grew on the anterior lip of the uterine cervix superficially. The stromal tissue was not torn by a tumor. In this case, the depth of invasion was 1.8 mm on the surgical specimen. **b**. Patient No. 8. On T2-weighted sagittal images, a flat tumor grew on the posterior lip of the cervix. The low intensity of cervical stroma was somewhat irregular. We diagnosed the lesion as invasive carcinoma. In this case, a depth of invasion was 3.1 mm on the surgical specimen. **c**. Patients No. 9. On T2-weigted sagittal images, the papillary tumor was detected on the cervical os. The stroma of the anterior and posterior lips was torn by a tumor. In this case, the depth of invasion was 9 mm on the surgical specimen.

MRI generally revealed significant stromal invasion of PSCC, if present. Seven (58%) patients exhibited no apparent masses or stromal invasion of PSCC on MRI before surgery (Additional file 3: Table S4). Three of seven patients actually displayed no stromal invasion on the surgical specimens. These seven cases ultimately involved stromal invasion with a depth ranging from 0 to 3 mm. However, the remaining five (42%) cases in which MRI demonstrated stromal invasion involved microscopic invasion ranging from 3.1 to 35 mm in depth. The two groups in which invasion was or was not detected on MRI did not overlap (Figure 4b). In this study, the border depth of microscopic invasion was approximately 3 mm, which was used to predict stromal invasion on MRI before surgery (Figure 5a-c).

With respect to the 12 true PSCC cases, the gross appearance of the lesion was papillary, wart-like, polypoid and exophytic, except for one case. A gross appearance of papillary, wart-like or polypoid lesions was considered to indicate the presence of ‘carcinoma’ in accordance with the FIGO staging (NCCN Guidelines, version 2.2013). Therefore, we diagnosed these cases as carcinoma over stage IB. In four of these cases, no tumor was apparent on MRI (No. 2, No. 3, No. 4 and No. 5) (Additional file 1: Table S1). The clinical diagnosis in these cases was IB1 stage; however, three cases involved a final pathological diagnosis of PSCC without stromal invasion (depth of invasion: 0 mm) (No. 2, No. 3 and No. 4). When we compared the clinical diagnoses with the post-operative diagnoses, we observed down staging in six (50%) of the 12 cases. Three lesions were downstaged due to a lack of stromal invasion and three due to the tumor size.

The treatments for PSCC are presented in Additional file 1: Table S1 and Additional file 3: Table S5. Conization followed by laparoscopic hysterectomy was performed in two (17%) cases, whereas radical surgery (radical hysterectomy or trachelectomy) was performed in the remaining 10 (83%) cases. The relationship between the surgical method and the final depth of tumor invasion is presented in Additional file 3: Table S5. According to the final pathological findings, seven (58%) of the 12 PSCC cases exhibited no stromal invasion (preinvasive carcinoma) or invasion less than or equal to 3 mm in depth/7 mm in horizontal spread (microinvasive carcinoma). Although four patients (No. 3, No. 4, No. 6, No. 7) fell within this category and showed no lymphovascular space invasion, they were treated with radical hysterectomy. All 10 patients treated with radical surgery had no lymph node metastases, and all 12 patients with PSCC exhibited no episodes of recurrence (mean: 49 months) and remain alive today.

Written informed consent was obtained from each patient for the publication of this research article and any accompanying images.

## Discussion

In 1952, Marsh reported 31 cases of papillary cervical lesions, among which, three were malignant [3]. In 1955, Kistner and Hertig suggested the malignant potential of cervical papilloma [4]. In 1958, Kazal reported 20 cases of papilloma of the cervix, two of which involved malignant transformation to squamous cell carcinoma [5]. In 1986, Randall first reported nine cases of PSCC and presented their criteria for distinguishing these lesions from squamous papilloma, condyloma and verrucous carcinoma [1]. Subsequently, Koenig divided PSCC into three groups histologically: predominantly squamous, predominantly transitional and mixed types [6]. The authors also concluded that the majority of these tumors were variants of squamous cell carcinoma of the cervix based on the findings of immunohistochemical analyses. In the current study, all 12 cases were predominantly squamous cell carcinoma or mixed type.

Papillary tumors growing on the uterine cervix are difficult to diagnose precisely. In this study, if the gross appearance revealed carcinomatous and papillary growth on the cervix, we diagnosed the lesion as invasive carcinoma based on the FIGO staging (NCCN Guidelines, version 2.2013). In addition, the colposcopic selective biopsies cannot be used to detect tumor stromal invasion. Therefore, different pathological diagnoses are sometimes recognized between the first colposcopic selective biopsy and the analysis of the resected surgical specimens. In this study, 28 cases were diagnosed as PSCC on a colposcopic selective biopsy; however, only 12 cases (43%) were true PSCC, whereas the others were non-keratinizing or microinvasive SCC (except for one case of adenosquamous carcinoma). We therefore experienced 16 cases of false PSCC. Moreover, we analyzed the surgical specimens microscopically, which involved assessing the degree of tumor heterogeneity. Consequentially, the surface components consisted of papillary growth, while the major deep components primarily comprised non-keratinizing SCC. Therefore, two or three colposcopic selective biopsies detected only superficial PSCC-like lesions. In Japan, an exact final diagnosis of PSCC is obtained using surgical specimens. In Europe and the USA, advanced cases of PSCC are diagnosed on the colposcopic selective biopsies and treated with radiation therapy and chemotherapy (NCCN Guidelines, version 2.2013). These cases may include conventional SCC tumors whose major component is SCC and a small portion of superficial lesions that are PSCC-like. In past studies, such cases were finally diagnosed as PSCC and correlated with a poor prognosis. The cases of false PSCC observed in this study involved lesions in more advanced stages (stage IB1 ~ IIB). This finding raises the following questions: Were the advanced PSCCs with a poor prognosis true PSCCs? Did they include a majority of components of conventional SCC? In cases of advanced PSCC treated with radiation therapy, deep biopsies should be performed to obtain the exact diagnosis before administering therapy.

The biological and clinical behavior of PSCC is not well understood. Mirhashemi et al. reported that the rate of positivity for high-risk HPV in cases of PSCC is generally less than that observed in cases of conventional SCC (50% vs >95%) [7, 8]. Therefore, it is possible that there are different mechanisms of carcinogenesis and clinical characteristics between PSCC and SCC. The authors also stated that eight (67%) of 12 PSCC cases exhibited a high proliferative activity, whereas five (83%) of six HPV-16-positive PSCC lesions demonstrated a high proliferative activity on Ki-67 immunostaining. Based on these biological data, PSCCs are proliferatively active tumors [9, 10]. However, Randall reported that two of nine patients developed recurrence more than seven years (87 and 106 months) after diagnosis [1], and Koenig also noted vaginal recurrence 12 years after the initial diagnosis [6]. Moreover, Mirhashemi et al. indicated a tendency toward a more indolent behavior of this tumor [7]. In their study, 10 of 12 patients were found to be free of disease and one patient continued to remain alive with disease, despite having a stage IIIA status. Indeed, none of our 12 patients with PSCC developed recurrence (mean: 49 months) and all are currently alive. We also suspect that the slow clinical behavior of PSCC is dissimilar to that of conventional SCC. In the present study, no invasive PSCC (IB1 ~ IIB) lesions were associated with lymph node metastasis (PSCC vs SCC: 0/11 (0%) vs 9/15 (60%)). We were unable to identify the etiology of the indolent behavior of PSCC; however, our findings suggest that true PSCC of the whole tumor is associated with a good prognosis.

Clinicians should select appropriate therapy for rare tumors, such as PSCCs. In this study, we evaluated the tumor depth before surgery using MRI. Due to the rarity of the disease, the MRI findings of PSCC have not yet been reported. In the current series, none of the four cases of PSCC without stromal invasion on the surgical specimens were detected as apparent tumors on MRI. Moreover, the cases with MRI findings of no apparent tumors or invasive lesions were ultimately found to involve stromal invasion ranging from 0 to 3 mm in depth. In other words, the cases in which MRI detected invasive tumors involved stromal invasion greater than 3 mm in depth. Although the number of PSCC cases in this study was small, we were able to evaluate the ability of MRI to detect stromal invasion. It has been previously reported that MRI can be used to detect deep invasion (more than 3 mm) of conventional cervical carcinomas, although this modality cannot clearly detect preinvasive disease (no stromal invasion) or microinvasive disease (stromal invasion less than or equal to 3 mm) [11]. These MRI data support our PSCC results. MRI can be applied to evaluate patients who desire fertility preservation therapy and may be excellent candidates.

## Conclusion

**Figure 6:**
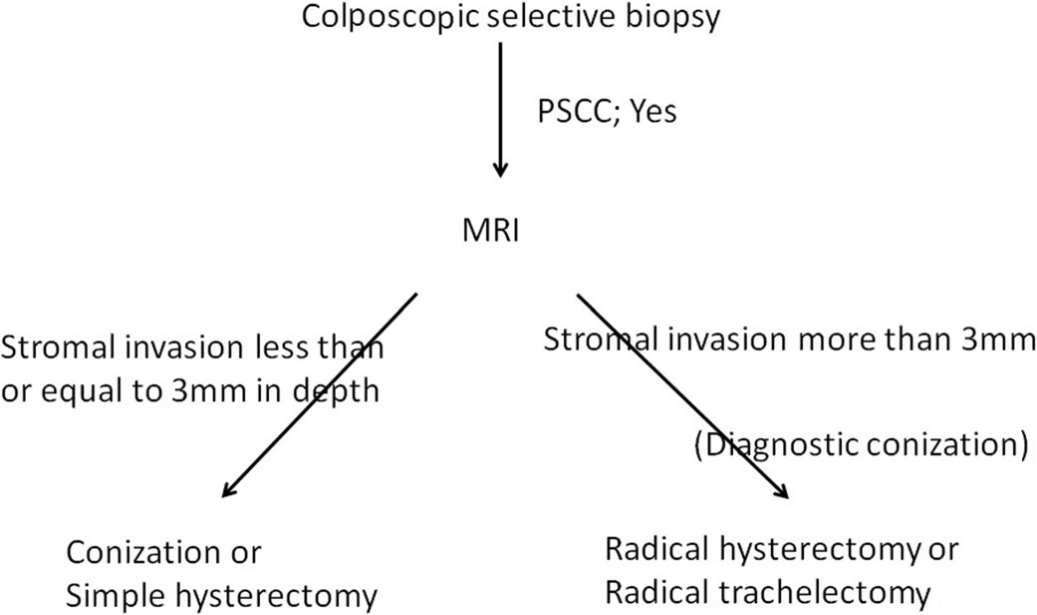
**An algorithm for the management of PSCC.**

In general, we perform the colposcopic selective biopsies in the outpatient clinic followed by conization to determine the method of treatment. It has been proposed that conization should be conducted to diagnose PSCC. However, it is impossible to perform conization in all patients with cervical cancer, and clinicians are usually unable to judge the presence or degree of stromal invasion with respect to PSCC. Therefore, MRI should first be performed to assess stromal invasion (>3 mm or ≦3 mm). If MRI does not detect tumor invasion, the depth of stromal invasion may range from 0 mm to 3 mm. We previously selected radical surgery for cases involving preinvasive and microinvasive disease. However, it is possible that minimally invasive surgery, such as conization ( for combined diagnosis and therapy) or laparoscopic hysterectomy, can be successfully used to treat preinvasive disease (no stromal invasion) or microinvasive disease (invasion less than or equal to 3 mm in depth/7 mm in horizontal spread) without inducing lymphovascular space invasion due to the indolent behavior and favorable prognosis of PSCC. If MRI reveals invasion of more than 3 mm, radical surgery should be selected according to guidelines. An algorithm for the management of PSCC is presented in Figure 6. In conclusion, the number of surgeries for fertility preservation therapy using minimally invasive procedures to treat PSCC can be increased.

## Electronic supplementary material

Additional file 1: Table S1: The clinicopathological data of the 12 true PSCC patients. (PPTX 85 KB)

Additional file 2: Table S2: The clinicopathological data of the 16 false PSCC patients. (PPTX 88 KB)

Additional file 3: Table S3: The relationship between the initial diagnosis of stromal invasion (with or without) on the colposcopic selective biopsy and the depth of invasion on the surgical specimen. **Table S4.** The relationship between the findings of stromal invasion (with or without) on MRI and the depth of invasion on the surgical specimen. **Table S5.** The relationship between the surgical method, the depth of invasion on the surgical specimen, and lymph node metastasis (with or without). (PPTX 77 KB)
