# Supplementary material for: Clinical approaches to treating papillary squamous cell carcinoma of the uterine cervix
Source: BMC Cancer. 2014 Oct 27;14:784. doi: 10.1186/1471-2407-14-784 (PMC4232646; doi:10.1186/1471-2407-14-784)
Supplement: Supplementary file 1 — Additional file 1: Table S1: The clinicopathological data of the 12 true PSCC patients. (PPTX 85 KB) [file 12885_2014_4983_MOESM1_ESM.pptx]

## Slide 1
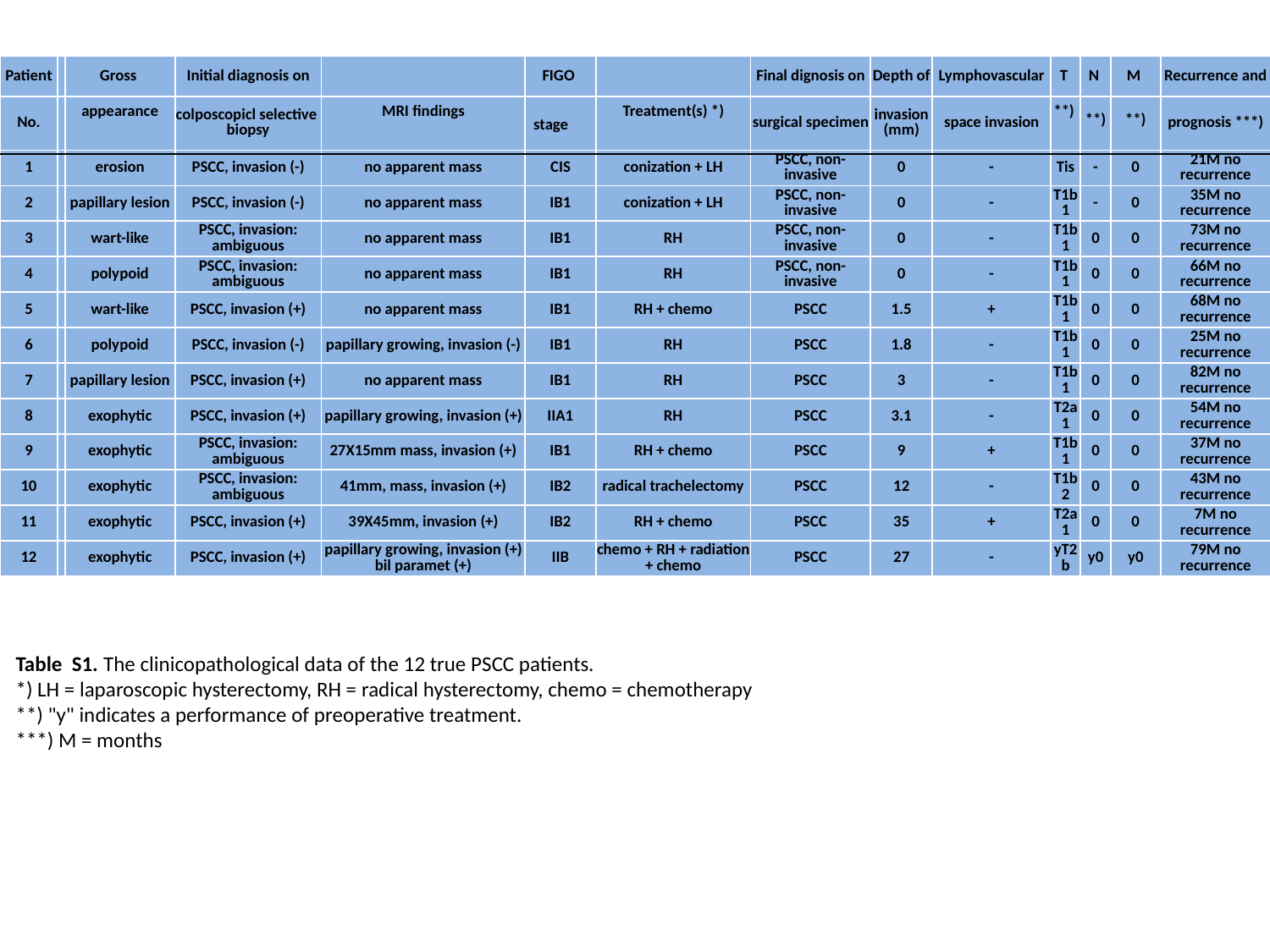

| Patient | | Gross | Initial diagnosis on | | FIGO | | Final dignosis on | Depth of | Lymphovascular | T | N | M | Recurrence and |
| --- | --- | --- | --- | --- | --- | --- | --- | --- | --- | --- | --- | --- | --- |
| No. | | appearance | colposcopicl selective biopsy | MRI findings | stage | Treatment(s) \*) | surgical specimen | invasion (mm) | space invasion | \*\*) | \*\*) | \*\*) | prognosis \*\*\*) |
| 1 | | erosion | PSCC, invasion (-) | no apparent mass | CIS | conization + LH | PSCC, non-invasive | 0 | - | Tis | - | 0 | 21M no recurrence |
| 2 | | papillary lesion | PSCC, invasion (-) | no apparent mass | IB1 | conization + LH | PSCC, non-invasive | 0 | - | T1b1 | - | 0 | 35M no recurrence |
| 3 | | wart-like | PSCC, invasion: ambiguous | no apparent mass | IB1 | RH | PSCC, non-invasive | 0 | - | T1b1 | 0 | 0 | 73M no recurrence |
| 4 | | polypoid | PSCC, invasion: ambiguous | no apparent mass | IB1 | RH | PSCC, non-invasive | 0 | - | T1b1 | 0 | 0 | 66M no recurrence |
| 5 | | wart-like | PSCC, invasion (+) | no apparent mass | IB1 | RH + chemo | PSCC | 1.5 | + | T1b1 | 0 | 0 | 68M no recurrence |
| 6 | | polypoid | PSCC, invasion (-) | papillary growing, invasion (-) | IB1 | RH | PSCC | 1.8 | - | T1b1 | 0 | 0 | 25M no recurrence |
| 7 | | papillary lesion | PSCC, invasion (+) | no apparent mass | IB1 | RH | PSCC | 3 | - | T1b1 | 0 | 0 | 82M no recurrence |
| 8 | | exophytic | PSCC, invasion (+) | papillary growing, invasion (+) | IIA1 | RH | PSCC | 3.1 | - | T2a1 | 0 | 0 | 54M no recurrence |
| 9 | | exophytic | PSCC, invasion: ambiguous | 27X15mm mass, invasion (+) | IB1 | RH + chemo | PSCC | 9 | + | T1b1 | 0 | 0 | 37M no recurrence |
| 10 | | exophytic | PSCC, invasion: ambiguous | 41mm, mass, invasion (+) | IB2 | radical trachelectomy | PSCC | 12 | - | T1b2 | 0 | 0 | 43M no recurrence |
| 11 | | exophytic | PSCC, invasion (+) | 39X45mm, invasion (+) | IB2 | RH + chemo | PSCC | 35 | + | T2a1 | 0 | 0 | 7M no recurrence |
| 12 | | exophytic | PSCC, invasion (+) | papillary growing, invasion (+) bil paramet (+) | IIB | chemo + RH + radiation + chemo | PSCC | 27 | - | yT2b | y0 | y0 | 79M no recurrence |
Table S1. The clinicopathological data of the 12 true PSCC patients.
*) LH = laparoscopic hysterectomy, RH = radical hysterectomy, chemo = chemotherapy
**) "y" indicates a performance of preoperative treatment.
***) M = months
