# Supplementary material for: Clinical approaches to treating papillary squamous cell carcinoma of the uterine cervix
Source: BMC Cancer. 2014 Oct 27;14:784. doi: 10.1186/1471-2407-14-784 (PMC4232646; doi:10.1186/1471-2407-14-784)
Supplement: Supplementary file 2 — Additional file 2: Table S2: The clinicopathological data of the 16 false PSCC patients. (PPTX 88 KB) [file 12885_2014_4983_MOESM2_ESM.pptx]

## Slide 1
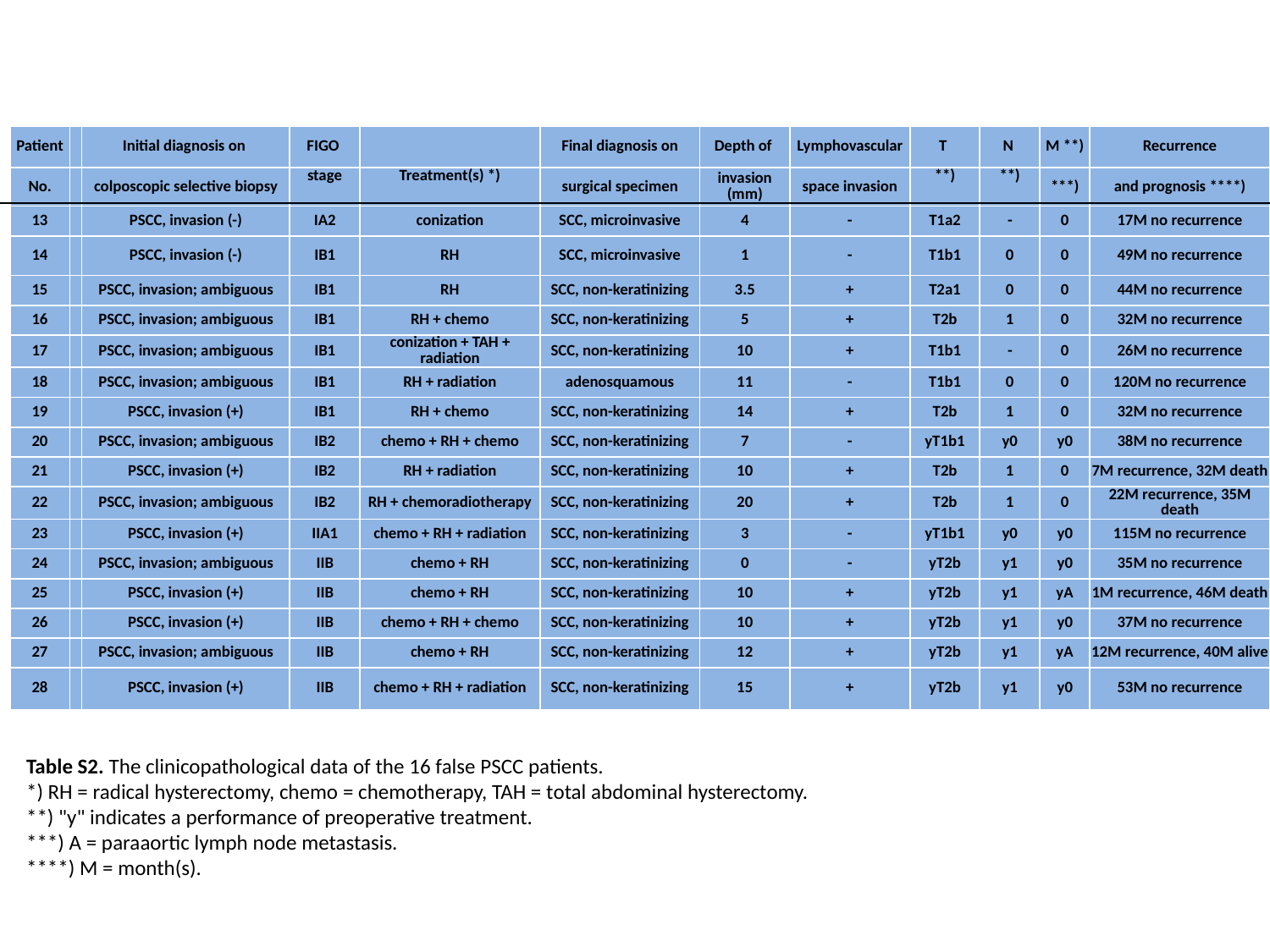

| Patient | | Initial diagnosis on | FIGO | | Final diagnosis on | Depth of | Lymphovascular | T | N | M \*\*) | Recurrence |
| --- | --- | --- | --- | --- | --- | --- | --- | --- | --- | --- | --- |
| No. | | colposcopic selective biopsy | stage | Treatment(s) \*) | surgical specimen | invasion (mm) | space invasion | \*\*) | \*\*) | \*\*\*) | and prognosis \*\*\*\*) |
| 13 | | PSCC, invasion (-) | IA2 | conization | SCC, microinvasive | 4 | - | T1a2 | - | 0 | 17M no recurrence |
| 14 | | PSCC, invasion (-) | IB1 | RH | SCC, microinvasive | 1 | - | T1b1 | 0 | 0 | 49M no recurrence |
| 15 | | PSCC, invasion; ambiguous | IB1 | RH | SCC, non-keratinizing | 3.5 | + | T2a1 | 0 | 0 | 44M no recurrence |
| 16 | | PSCC, invasion; ambiguous | IB1 | RH + chemo | SCC, non-keratinizing | 5 | + | T2b | 1 | 0 | 32M no recurrence |
| 17 | | PSCC, invasion; ambiguous | IB1 | conization + TAH + radiation | SCC, non-keratinizing | 10 | + | T1b1 | - | 0 | 26M no recurrence |
| 18 | | PSCC, invasion; ambiguous | IB1 | RH + radiation | adenosquamous | 11 | - | T1b1 | 0 | 0 | 120M no recurrence |
| 19 | | PSCC, invasion (+) | IB1 | RH + chemo | SCC, non-keratinizing | 14 | + | T2b | 1 | 0 | 32M no recurrence |
| 20 | | PSCC, invasion; ambiguous | IB2 | chemo + RH + chemo | SCC, non-keratinizing | 7 | - | yT1b1 | y0 | y0 | 38M no recurrence |
| 21 | | PSCC, invasion (+) | IB2 | RH + radiation | SCC, non-keratinizing | 10 | + | T2b | 1 | 0 | 7M recurrence, 32M death |
| 22 | | PSCC, invasion; ambiguous | IB2 | RH + chemoradiotherapy | SCC, non-keratinizing | 20 | + | T2b | 1 | 0 | 22M recurrence, 35M death |
| 23 | | PSCC, invasion (+) | IIA1 | chemo + RH + radiation | SCC, non-keratinizing | 3 | - | yT1b1 | y0 | y0 | 115M no recurrence |
| 24 | | PSCC, invasion; ambiguous | IIB | chemo + RH | SCC, non-keratinizing | 0 | - | yT2b | y1 | y0 | 35M no recurrence |
| 25 | | PSCC, invasion (+) | IIB | chemo + RH | SCC, non-keratinizing | 10 | + | yT2b | y1 | yA | 1M recurrence, 46M death |
| 26 | | PSCC, invasion (+) | IIB | chemo + RH + chemo | SCC, non-keratinizing | 10 | + | yT2b | y1 | y0 | 37M no recurrence |
| 27 | | PSCC, invasion; ambiguous | IIB | chemo + RH | SCC, non-keratinizing | 12 | + | yT2b | y1 | yA | 12M recurrence, 40M alive |
| 28 | | PSCC, invasion (+) | IIB | chemo + RH + radiation | SCC, non-keratinizing | 15 | + | yT2b | y1 | y0 | 53M no recurrence |
Table S2. The clinicopathological data of the 16 false PSCC patients.
*) RH = radical hysterectomy, chemo = chemotherapy, TAH = total abdominal hysterectomy.
**) "y" indicates a performance of preoperative treatment.
***) A = paraaortic lymph node metastasis.
****) M = month(s).
