# Supplementary material for: Clinical approaches to treating papillary squamous cell carcinoma of the uterine cervix
Source: BMC Cancer. 2014 Oct 27;14:784. doi: 10.1186/1471-2407-14-784 (PMC4232646; doi:10.1186/1471-2407-14-784)
Supplement: Supplementary file 3 — Additional file 3: Table S3: The relationship between the initial diagnosis of stromal invasion (with or without) on the colposcopic selective biopsy and the depth of invasion on the surgical specimen. Table S4. The relationship between the findings of stromal invasion (with or without) on MRI and the depth of invasion on the surgical specimen. Table S5. The relationship between the surgical method, the depth of invasion on the surgical specimen, and lymph node metastasis (with or without). (PPTX 77 KB) [file 12885_2014_4983_MOESM3_ESM.pptx]

## Slide 1
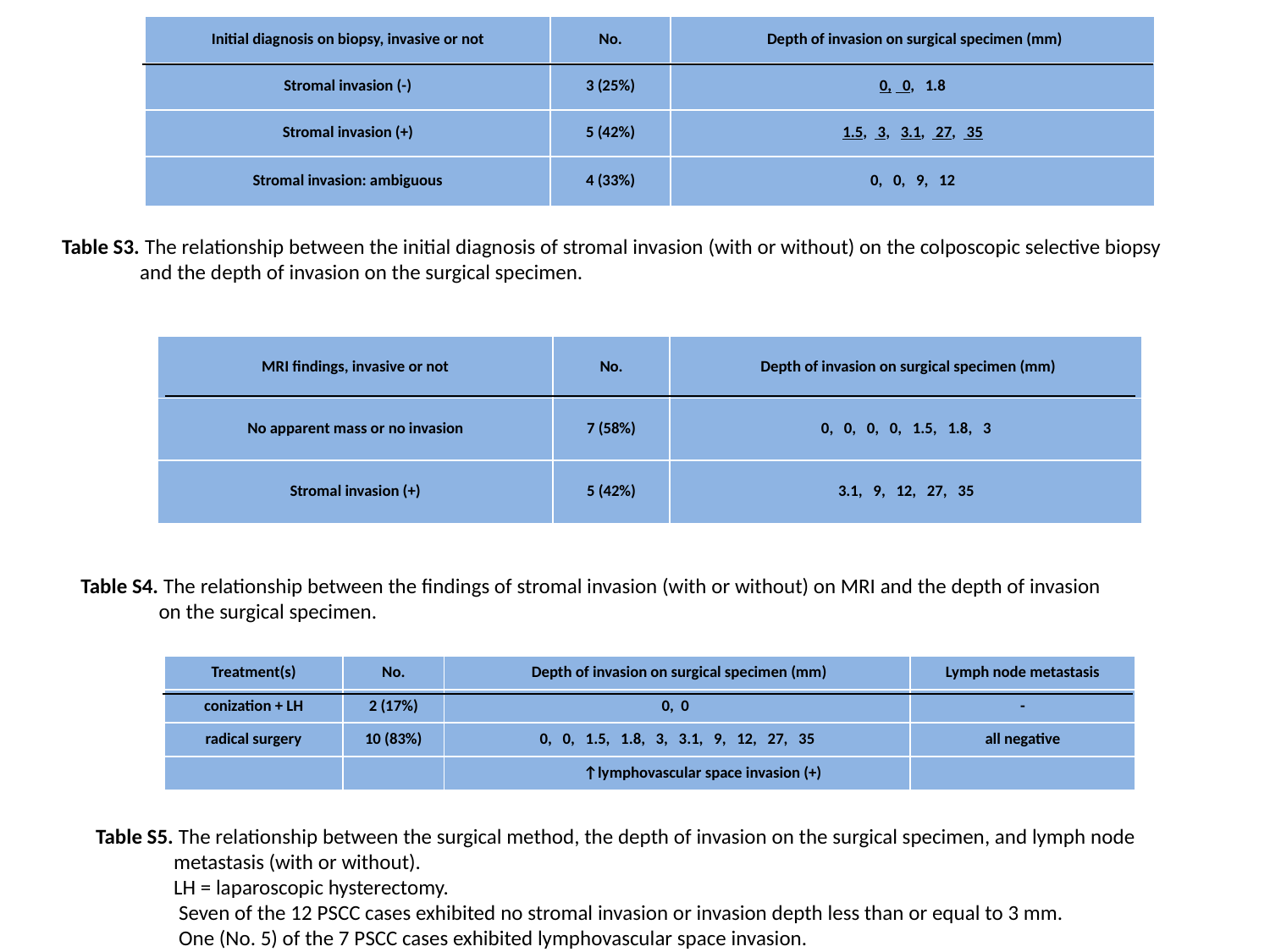

| Initial diagnosis on biopsy, invasive or not | No. | Depth of invasion on surgical specimen (mm) |
| --- | --- | --- |
| Stromal invasion (-) | 3 (25%) | 0, 0, 1.8 |
| Stromal invasion (+) | 5 (42%) | 1.5, 3, 3.1, 27, 35 |
| Stromal invasion: ambiguous | 4 (33%) | 0, 0, 9, 12 |
Table S3. The relationship between the initial diagnosis of stromal invasion (with or without) on the colposcopic selective biopsy
 and the depth of invasion on the surgical specimen.
| MRI findings, invasive or not | No. | Depth of invasion on surgical specimen (mm) |
| --- | --- | --- |
| No apparent mass or no invasion | 7 (58%) | 0, 0, 0, 0, 1.5, 1.8, 3 |
| Stromal invasion (+) | 5 (42%) | 3.1, 9, 12, 27, 35 |
Table S4. The relationship between the findings of stromal invasion (with or without) on MRI and the depth of invasion
 on the surgical specimen.
| Treatment(s) | No. | Depth of invasion on surgical specimen (mm) | Lymph node metastasis |
| --- | --- | --- | --- |
| conization + LH | 2 (17%) | 0, 0 | - |
| radical surgery | 10 (83%) | 0, 0, 1.5, 1.8, 3, 3.1, 9, 12, 27, 35 | all negative |
| | | ↑lymphovascular space invasion (+) | |
Table S5. The relationship between the surgical method, the depth of invasion on the surgical specimen, and lymph node
 metastasis (with or without).
 LH = laparoscopic hysterectomy.
 Seven of the 12 PSCC cases exhibited no stromal invasion or invasion depth less than or equal to 3 mm.
 One (No. 5) of the 7 PSCC cases exhibited lymphovascular space invasion.
